# The 5′-end segment-specific noncoding region of influenza A virus regulates both competitive multi-segment RNA transcription and selective genome packaging during infection

**DOI:** 10.1128/jvi.00328-25

**Published:** 2025-08-05

**Authors:** Zining Liu, Lei Zhang, Wenyu Zhang, Yuerong Lai, Tao Deng

**Affiliations:** 1 Laboratory of Pathogen Microbiology and Immunology, Institute of Microbiology, Chinese Academy of Sciences 85387, Beijing, China; 2 Medical School, University of the Chinese Academy of Sciences 74519, Beijing, China; 3 Institute of Pediatrics, Shenzhen Children’s Hospital, Shenzhen, China; St. Jude Children's Research Hospital, Memphis, Tennessee, USA

**Keywords:** influenza A virus, 5′-end H1-ssNCRs, competitive viral RNA transcription, selective genome packaging

## Abstract

The 3′ and 5′ termini of the eight RNA segments of the influenza A virus (IAV) genome contain segment-specific or subtype-specific (in HA and NA segments) noncoding regions (ssNCRs), located between the highly conserved terminal promoter sequences and the coding start or stop codons. These ssNCRs vary significantly in both length and sequence among segments and have been implicated in selective genome packaging. Beyond their established role in packaging, we previously demonstrated that the 3′-end H1-ssNCR, together with its adjacent coding region, supports optimal replication of HA viral RNA (vRNA) in a multi-segment context. In this study, we discovered that truncation of the 5′-end H1-ssNCR, unlike its 3′ counterpart, resulted in a reduction in HA mRNA levels in a template-competitive manner and impaired incorporation of HA vRNA into progeny virions. Interestingly, upon passaging of a recombinant virus carrying the most severely truncated 5′-end H1-ssNCR, an adaptive mutation occurred seven nucleotides upstream of the truncation site. This mutation restored both HA mRNA expression and HA vRNA packaging, thereby rescuing viral replication. Further analysis revealed that these regulatory effects may be mediated by RNA secondary structures formed around the 5′-end H1-ssNCR. Collectively, our findings reveal, for the first time, a dual regulatory role of the 5′-end segment-specific noncoding regions of influenza A virus RNA segments in modulating multi-segmental RNA transcription and selective genome packaging during infection.

**IMPORTANCE:**

The 3′ and 5′ segment-specific noncoding regions (ssNCRs) of influenza A virus (IAV) have long been recognized as critical signals for selective genome packaging. However, their potential roles in other regulatory processes remain largely unexplored. We previously reported that the 3′-end H1-ssNCR, together with its adjacent coding region, primarily determines optimal HA vRNA accumulation in a multi-segment environment. In this study, we further demonstrate that the 5′-end ssNCR plays a dual role in regulating viral RNA transcription in a template-competitive manner and governing vRNA incorporation into progeny virions. These findings reveal previously underappreciated levels of complexity, highlighting that ssNCRs contribute not only to genome packaging but also to the fine-tuning of viral RNA synthesis in multi-segmented negative-strand RNA viruses.

## INTRODUCTION

Influenza A viruses (IAVs) are major pathogens leading to annual epidemics and occasional pandemics worldwide (1). IAVs belong to the family Orthomyxoviridae that is characterized by their segmented, negative-sense, single-stranded RNA genome. The eight-RNA segment genome of IAV is composed of bilateral 3′ and 5′ noncoding regions and central open reading frames (ORFs) in negative sense. The 3′ and 5′ noncoding regions are composed of the highly conserved promoter regions (12 nucleotides at the 3′ end and 13 nucleotides at the 5′ end) among all influenza A viruses and segment-specific or subtype-specific (in HA and NA segments) noncoding regions (ssNCRs) which are conserved in the same vRNA segment of different influenza A viruses (2). These ssNCRs vary significantly in both length and sequence at the 3′ and 5′ ends among the different segments or different HA and NA subtype segments (3).

The eight segments, encoding at least ten major viral proteins (PB2, PB1, PA, HA, NP, NA, M1, M2, NS1, and NS2), are incorporated into virions in the form of viral ribonucleoprotein (vRNP) complexes (4). Within each vRNP, the highly conserved promoter regions, together with their adjacent 2–3 segment-specific nucleotides, join the 3′ and 5′ ends together by 2–3 Watson-Crick base pairs forming into a special structure in which a 5´ hook structure plays a critical role in specifically binding to the heterotrimeric viral RNA-dependent RNA polymerase complex (RdRp) (composed by PB2, PB1, and PA) (5). The rest of RNA is bound to the two nucleoprotein (NP) strands of opposite polarity, resulting in a double helical conformation of vRNPs (6). The vRNP is the minimal functional unit required for viral RNA transcription and replication to occur in the nucleus of infected cells (7).

Viral RNA transcription is the process of the vRNA-directed mRNA synthesis. The initiation of transcription requires viral RdRp to intimately associate with the C-terminal domain of host RNA polymerase II (Pol-II-CTD) to facilitate RdRp to snatch capped RNAs from host pre-mRNAs and then use them as initiating primers (8). The elongation of the mRNA transcription is pre-terminated when the resident RdRp reaches the 5′ hook of the vRNA and stopped by steric hindrance (9). The RdRp then reiterates copying a stretch of U close to the 5′ hook, leading to the polyadenylation of the mRNA (9, 10). In contrast, viral RNA replication is a process of two steps (vRNA to cRNA and then cRNA to vRNA) with different initiation strategies (11). Both steps are full length copying of the templates. The structural studies have revealed that, upon binding to different ligands (e.g., vRNA/cRNA promoters, Pol II-CTD, and host ANP32A), viral RdRp forms quite different conformations to fulfill the viral RNA transcription, replication, and encapsidation (12, 13). Furthermore, during a viral infection cycle, the viral RNA transcription and replication are tightly controlled and the expressions of different viral proteins exhibit distinct patterns in both quantity and timing (14, 15).

Newly synthesized vRNPs are exported from the nucleus and subsequently trafficked through the cytoplasm to the plasma membrane, where they are selectively packaged into progeny virions. Each vRNA segment contains distinct packaging signals that span both the segment-specific noncoding regions and adjacent coding sequences, with lengths varying across different segments (16). Studies have shown that the spacing between NP binding sites along the RNA leaves substantial stretches of RNA exposed, allowing for potential intra- and intersegment RNA-RNA interactions (17, 18). Notably, the NP associates with vRNA in a non-uniform and non-random manner (19). This uneven arrangement results in NP-free regions on the vRNP surface that can form flexible RNA secondary and/or tertiary structures, which are thought to play essential roles in mediating the selective packaging of the viral genome (20, 21).

It has been reported that the 3′ and 5′ noncoding regions, together with terminal coding regions, act as packaging signals for selective packaging of the eight vRNPs into budding viral particles (22–24). Especially, it has been proposed that the 3′ and 5′-end ssNCRs serve as virion incorporation signals and the terminal coding regions serve as a bundling signal (25). In addition, the 3′ and 5′-end ssNCRs have been implicated in regulating viral RNA transcription and replication (26). We previously found that no stringent compatibility between the 3′ and 5′ ends of H1-ssNCRs is required for efficient viral multiplication (27). Furthermore, through serial truncations of the 3′-end H1-ssNCR, we found that a synergistic effect between the 3′-end H1-ssNCR and its adjacent coding region is mainly involved in regulating the optimal HA vRNA levels in a multi-segment environment (28).

In this study, we investigated the functional roles of the 5′-end ssNCR of the H1 subtype-segment using a series of truncation mutagenesis experiments. In addition to its known function in acting as a packaging signal, interestingly, we found that extensive truncation of the 5′-end H1-ssNCR especially resulted in decreased H1-vRNA transcription but not replication under multiple-template conditions. During serial passaging of the most attenuated truncation mutant, a compensatory (revertant) mutation emerged just seven nucleotides upstream of the truncation site. This mutation restored HA mRNA expression, HA vRNA incorporation into virions, and overall virus replication. Further analysis suggests that RNA secondary structures surrounding the 5′-end ssNCRs may mediate template-competitive transcription and selective packaging processes. Together, our results propose a model in which the 5′-end ssNCRs play a dual role in fine-tuning optimal viral mRNA transcription and ensuring maximal vRNA incorporation during influenza A virus replication.

## RESULTS

### The truncations of the 5′-end H1-ssNCR result in virus attenuation

**Fig 1:**
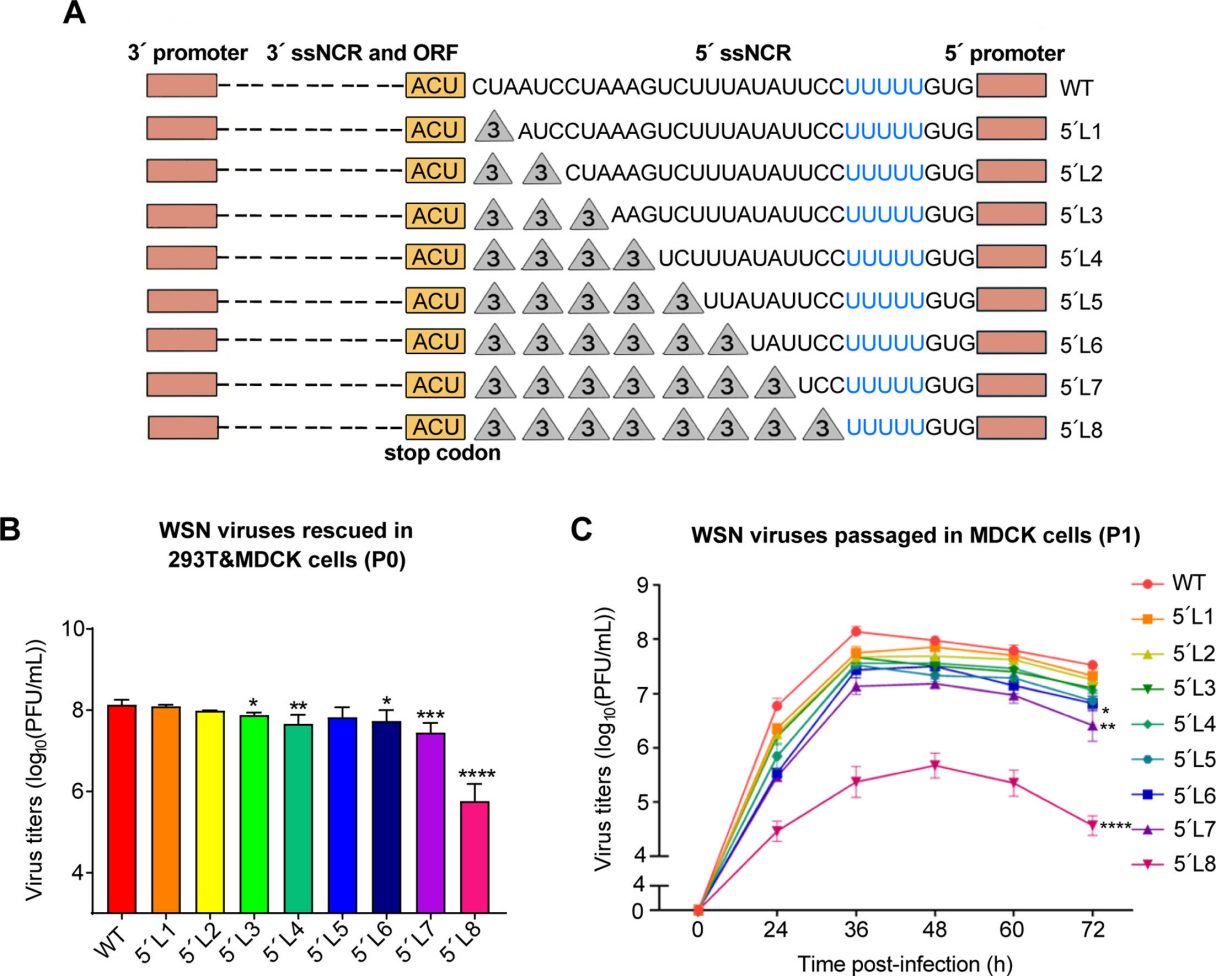
The truncations of the 5′-end H1-ssNCR result in virus attenuation. (**A**) Schematic representation of mutants with serial truncations at the 5′-end H1-ssNCR of the HA segment of the WSN virus. The pink boxes indicate the 3′- and 5′-terminal promoter regions. The orange boxes denote the stop codon (UGA) of HA vRNA (ACU in negative sense). The dotted lines represent the 3′-end H1-ssNCR and the open reading frames, while the gray triangles indicate deleted nucleotides. Truncation was performed in three-nucleotide increments. The U stretch next to the 5′-end H1-ssNCR is labeled in blue. (**B**) Titers of WSN rescued viruses (P0). Wild-type or mutant viruses were rescued in HEK-293T and MDCK co-cultured cells by co-transfecting eight pHW2000-PB2, -PB1, -PA, mut-HA (wild-type or truncation), -NP, -NA, -M, and -NS plasmids. The graph displays the mean titers of wild-type and mutant viruses in P0. Statistical significance was determined using two-tailed Student´s t test; asterisks indicate significant differences from the wild-type virus as follows: *, *P* ＜0.05; **, *P* ＜0.01; ***, *P* ＜0.001; ****, *P* ＜0.0001. (**C**) Growth curves of WSN wild-type and mutant viruses infected at an MOI of 0.001 (P1). Viral growth curves were determined by plaque assay on MDCK cells at 24 h, 36 h, 48 h, 60 h, and 72 h post-infection. Statistical significance was determined using a two-way analysis of variance with Dunnett correction for multiple comparisons; asterisks indicate significant differences from wild-type virus as follows: *, *P* ＜0.05; **, *P* ＜0.01; ****, *P* ＜0.0001. The data in panels **B** and **C** represent the means ± SEM from three independent experiments.

To assess the importance of the 5′-end H1-ssNCR for IAV replication, we first generated eight 5′-end H1-ssNCR truncation mutants in the eight-plasmid reverse genetic system of influenza A/WSN/33 (H1N1) virus. Specifically, we progressively truncated the 5′-end H1-ssNCR by deleting three nucleotides at a time from downstream of the HA stop codon to upstream of the U stretch, resulting in eight mutants (from 5′L1 to 5′L8) with 3, 6, 9, 12, 15, 18, 21, and 24 nt deletions (Fig. 1A).

Next, we used reverse genetics to generate recombinant WSN mutant viruses containing the serial 5′-end H1-ssNCR truncations and examined the titers of the rescued viruses (passage 0, P0) by plaque assay. As shown in Fig. 1B, the 5′L1 and 5′L2 viruses showed similar titers to the wild-type virus and the 5′L3–5′L7 viruses showed mild titer reductions within the range of 1 log. In contrast, the 5′L8 virus showed drastic reduction in titers (> 2 log) (Fig. 1B). We then examined the growth curves of all these rescued mutant viruses at passage 1 (P1) in MDCK cells. As shown in Fig. 1C, similar to the titers observed at P0 (Fig. 1B), 5′L1 to 5′L7 showed mild reduction in growth, while 5′L8, the entire 5′-end H1-ssNCR truncation between stop codon and the U stretch, showed severe growth reduction. These results suggest that WSN virus tolerates most of the truncations in the 5′-end H1-ssNCR to a certain degree, but retaining the three nucleotides next to the U stretch is essential for virus multiplication.

### The truncation of the 5′-end H1-ssNCR between the stop codon and the U stretch significantly decreases HA vRNA incorporation and HA protein expression

**Fig 2:**
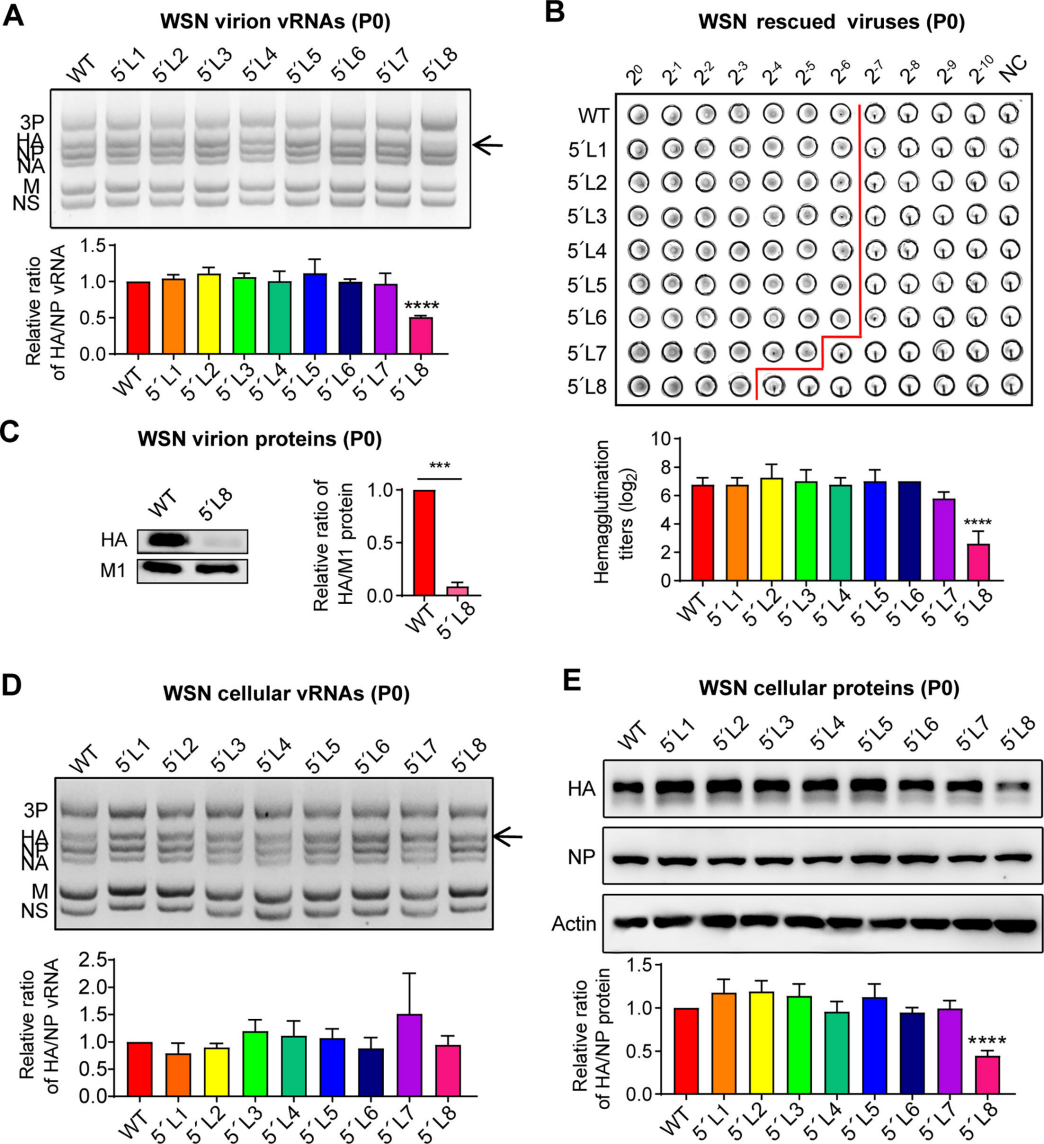
The truncation of the 5′-end H1-ssNCR between the stop codon and the U stretch significantly decreases both HA vRNA incorporation and HA protein expression. (**A–C**) Wild-type or mutant viruses were rescued in HEK-293T and MDCK co-cultured cells. Supernatants were harvested at 48 h post-transfection. (**A**) M-RT-PCR analysis of WSN wild-type and mutant vRNA levels in supernatants (P0). The graph displays the mean intensity signals of mutant HA/NP vRNAs relative to those of the wild-type. (**B**) Hemagglutination assay of WSN wild-type and mutant rescued viruses (P0). The graph shows the mean hemagglutination titers of mutant viruses relative to those of the wild-type virus. (**C**) Western blot analysis of WSN wild-type and mutant protein levels in supernatants (P0). The graph displays the mean intensity signals of the mutant HA/M1 protein relative to those of the wild-type. Statistical significance was determined using two-tailed Student´s t test; asterisks indicate significant differences from the wild-type virus as follows: ***, *P* ＜0.001. (**D, E**) Wild-type or mutant viruses were rescued in HEK-293T and MDCK co-cultured cells in the medium with 5 mM NH_4_Cl. Cells were harvested at 48 h post-transfection. (**D**) M-RT-PCR analysis of WSN wild-type and mutant vRNA levels in cells (P0). The graph displays the mean intensity signals of mutant HA/NP vRNAs relative to those of the wild-type. (**E**) Western blot analysis of WSN wild-type and mutant HA protein levels in cells (P0). The graph displays the mean intensity signals of mutant HA/NP protein relative to those of the wild-type. The data in panels **A** to **E** represent the means ± SEM from three independent experiments. Statistical significance for panels A, B, D, and was determined using one-way analysis of variance (ANOVA) with Dunnett corrections. Asterisks indicate significant differences from the wild-type template as follows: ****, *P* ＜0.0001.

To investigate the molecular basis of the attenuation in 5′-end H1-ssNCR truncated mutant viruses, we analyzed vRNA and protein levels of HA in the rescued virions harvested at 48 h post-transfection (P0). A multi-segment reverse transcription-PCR (M-RT-PCR) approach, which simultaneously amplifies all eight genomic RNA segments, was employed to detect vRNAs in the virions (29). The HA protein levels in harvested virions were analyzed using hemagglutination assays and Western blotting. As expected, the HA vRNA levels of the 5′L1 to 5′L7 mutants were comparable to those of the wild-type virus in rescued virions, whereas the 5′L8 mutant showed significantly reduced HA vRNA levels (Fig. 2A). Interestingly, we found that the hemagglutination titer of the 5′L8 mutant was also dramatically decreased compared to those of wild-type and 5′L1 to 5′L7 mutant virions (Fig. 2B). To determine whether the reduced hemagglutination titer was due to a lower number of virions or decreased HA protein incorporation into virions, we conducted Western blot analysis of HA and M1 (as an internal control) protein levels in both wild-type and 5′L8 virions. Notably, the ratio of HA to M1 levels in 5´L8 virions was dramatically lower than that in wild-type virions (Fig. 2C), suggesting that the truncation significantly reduces HA protein incorporation into virus particles.

To further explore the cause of reduced HA vRNA and HA protein levels in 5′L8 virions, we conducted a single-cycle rescue experiment in which cells were treated with 5 mM NH_4_Cl at 6 hours post-transfection to prevent re-entry of rescued virions. Harvested cells were analyzed using M-RT-PCR and Western blot analysis to detect HA vRNA and protein levels, respectively. Interestingly, the HA vRNA levels in transfected cells for the all truncation mutants including 5′L8 were all comparable to that of the wild-type (Fig. 2D). In contrast, HA protein levels in cells transfected with the 5′L8 mutant were significantly reduced (Fig. 2E). These findings suggest that the most severely truncated 5′-end H1-ssNCR does not impair HA vRNA accumulation but affects its incorporation into virions. Additionally, the truncation mutant led to significantly reduced HA protein accumulation in cells, which likely contributed to the lower levels of HA protein observed in virions. Collectively, these results strongly suggest that the 5′-end H1-ssNCRs play a key role in regulating both HA vRNA packaging into virion and HA protein accumulation in host cells.

### The truncation of the 5′-end ssNCRs between the U stretch and stop codon affects viral mRNA levels in a multi-template RNP reconstitution system

**Fig 3:**
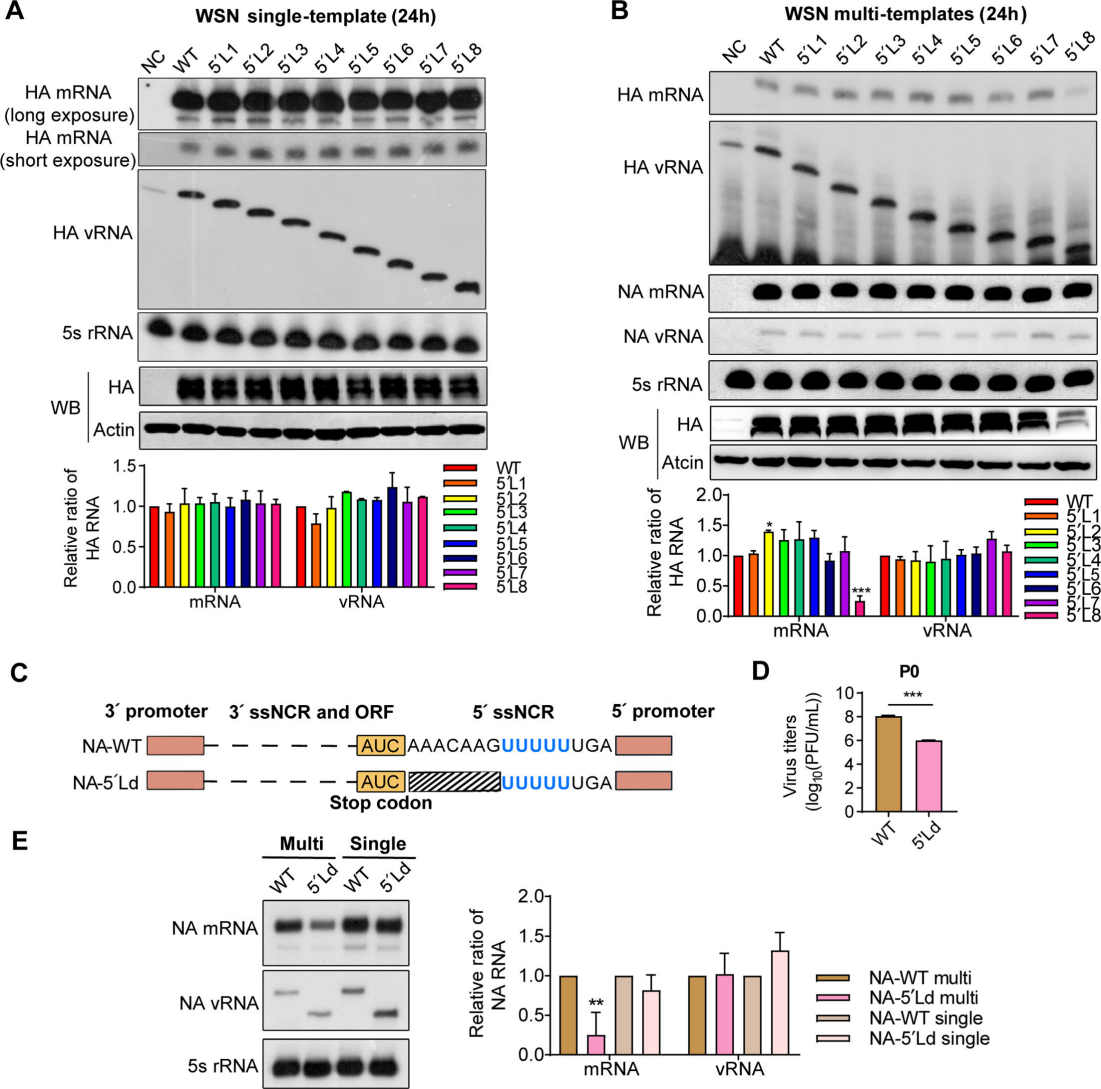
The truncation of the 5′-end ssNCR affects viral mRNA levels in a multi-template RNP reconstitution system. (**A**) Truncation of the 5′-end H1-ssNCR in the WSN single-template RNP reconstitution system. HEK-293T cells were co-transfected with plasmids expressing WSN viral polymerase subunits (PB2, PB1, and PA), NP, and either wild-type or truncation HA vRNA. (**B**) Truncation of the 5´-end H1-ssNCR in the WSN multi-template RNP reconstitution system. Seven pHW2000-derived plasmids expressing PB2, PB1, PA, mut-HA (wild-type or truncation), NP, NA, and NS of WSN virus were co-transfected into HEK-293T cells. In both **A** and **B**, polymerase containing active site mutations (PB1a, D445A/446A) were used as a negative control (NC). Accumulation of HA RNA at 24 h post-transfection was detected by primer extension analysis (top), while HA protein levels were assessed by Western blotting (bottom). The graphs display the mean signal intensity of mutant HA RNAs relative to wild-type levels. Statistical significance for panels **A** and **B** was determined using an one-way analysis of variance (ANOVA) with Dunnett correction for multiple comparisons; asterisks indicate significant differences from the wild-type template as follows: ***, *P* ＜0.001. (**C**) Truncation of the 5′-end NA-ssNCR in the single- and multi-template RNP reconstitution systems of WSN virus. The schematic depicts a mutant with the complete 5´-end NA-ssNCR truncated in the NA segment (NA-5´Ld), aligned with the wild-type NA of the WSN virus. The color-coded boxes and dotted lines denote the NA segment regions that correspond to the positions previously identified in the HA segment in Fig. 1A. (**D**) Titers of WSN rescued viruses (P0). The plasmids co-transfected into HEK-293T and MDCK mixed cells were the same as those in Fig. 1B, with the exception that the pHW-mut-HA plasmid was replaced with pHW-mut-NA (wild-type or NA-5′Ld). The graph displays the mean titers of wild-type and mutant viruses in P0. Statistical significance was determined using two-tailed Student´s t test; asterisks indicate significant differences from the wild-type virus as follows: ***, *P* ＜0.001. (**E**) Truncation of the 5′-end H1-ssNCR in the WSN single-template and multi-template RNP reconstitution system. The plasmids were co-transfected into HEK-293T cells, and analysis of harvested cells was performed as described for **A** and **B**, except that the pHW-mut-HA plasmid was replaced with pHW-mut-NA (wild-type or NA-5′Ld). The graph displays the mean signal intensity of mutant NA RNAs relative to those of the wild-type. Statistical significance was determined using a two-way analysis of variance (ANOVA) with Dunnett’s correction for multiple comparisons; asterisks indicate significant differences from the wild-type template as follows: **, *P* ＜0.01. The data in panels **A** to **E** represent the means ± SEM from three independent experiments.

Since the packaging role of segment-specific NCRs is widely accepted, we next focused on investigating the role of 5′-end H1-ssNCR in regulating viral protein accumulation in host cells. To this end, we used a vRNP reconstitution system of WSN virus. Human HEK-293T cells were co-transfected with four protein-expressing plasmids encoding the three viral polymerase subunits (PB2, PB1, and PA) and the nucleoprotein, along with a modified bidirectional pHW2000 plasmid in which the CMV promoter was inactivated (pHW-mut), allowing the plasmid expressing either wild-type or 5′-end H1-ssNCR truncated HA vRNAs only. At 24 hours post-transfection, steady-state levels of HA mRNA and vRNA were analyzed by primer extension analysis. A catalytically inactive polymerase (PB1a) was used as the negative control (30). As shown in Fig. 3A, all 5′-end H1-ssNCR truncated HA vRNAs were efficiently transcribed and replicated by the viral polymerase to levels comparable to that of the wild-type HA vRNA. Correspondingly, HA protein expression from these vRNAs was also similar to the wild-type level. These results suggest that the terminal 5′ noncoding nucleotides upstream the U stretch (as shown in the 5′L8 mutant) are dispensable for viral RNA transcription and replication in a single-template RNP reconstitution system.

Since we observed significantly reduced HA protein levels in both virions and transfected cells during the rescue experiments (Fig. 2C and E), we further investigated the effects of 5′-end ssNCR truncations using a multi-segment vRNP reconstitution system. In this system, seven pHW2000 plasmids (PB2, PB1, PA, NP, wild-type or truncation HA (without the CMV promoter), NA, and NS segments) from the WSN reverse genetic system were co-transfected into human HEK-293T cells. The pHW2000-M plasmid was excluded to avoid the formation of virus particles (28). We then assessed the accumulations of HA mRNA, vRNA, and protein. As shown in Fig. 3B, the 5′L8 mutant exhibited dramatically reduced steady-state levels of HA mRNA and HA protein compared to the wild-type and other truncation mutants. However, vRNA levels remained comparable across all templates. These results suggest that in a multi-template context, the most severe 5′-end ssNCR truncation (5′L8) impairs mRNA synthesis and HA protein accumulation, despite not affecting vRNA replication.

To explore whether this observation is applicable to other segments, we conducted a similar experiment using the NA segment. An H1 5′L8-equavalent mutant of the N1 segment (NA-5′Ld), which contains a 7-nucleotide truncation between the U stretch and stop codon (7 nt) of WSN virus (Fig. 3C), was generated to evaluate its effect on the virus titer and RNA synthesis in single- and multi-template vRNP reconstitution systems. As shown in Fig. 3D, the NA-5′Ld mutant virus (P0) exhibited a > 2 log reduction in the viral titer compared to the wild-type virus, consistent with the phenotype observed for the HA 5′L8 virus. In the single-template system, both NA mRNA and vRNA levels were comparable to those of the wild-type (Fig. 3E). However, in the multi-segment system, the NA-5′Ld mutant showed a marked reduction in mRNA levels, while vRNA levels remained unaffected (Fig. 3E). Taken together, these results further support the notion that the 5′-end ssNCRs of IAV contribute to the regulation of optimal mRNA accumulation in a multi-segment viral genome context.

### The 5′-end ssNCR affects viral mRNA levels in a template-competitive manner

**Fig 4:**
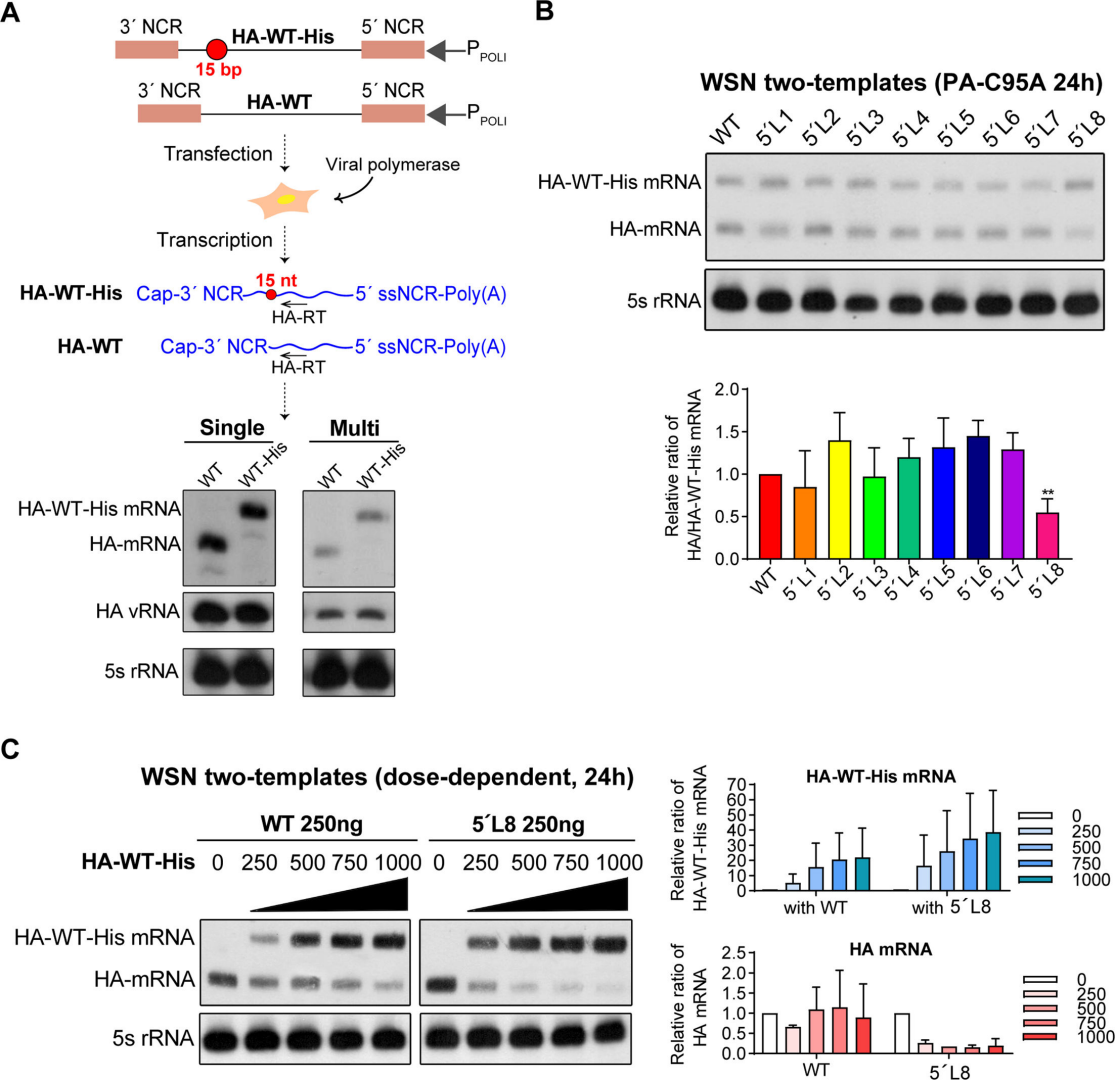
The 5′-end ssNCR affects viral mRNA levels in a template-competitive manner. (**A**) The schematic representation of the wild-type (HA-WT) and a modified wild-type HA template (HA-WT-His) which contains a 5 × His tag ORF insertion (15 nt) of the HA segment in the RNP reconstitution system. Both templates were derived from mutant vRNA-expressing plasmid (pHW-mut-HA). Pink boxes denote the 3´ and 5´ NCR of the HA segment, and black lines indicate the HA open reading frames. The 15 nt 5 × His tag is depicted in red circles. The mRNAs derived from transfected plasmids are shown as blue curly lines. Primer extension analysis shows the RNA levels of HA-WT or HA-WT-His in the single-template and multi-template RNP reconstitution systems. (**B**) Truncation of the 5′-end H1-ssNCR in a two-template competition RNP reconstitution system derived from the WSN virus. HEK-293T cells were co-transfected with pcDNA-PB2, pcDNA-PB1, pcDNA-PA-C95A, pcDNA-NP, pHW-mut-HA-WT-His, and pHW-mut-HA-WT/truncation plasmids. Accumulation of the HA mRNA at 24 h post-transfection was detected by primer extension analysis. The graph displays the mean signal intensity of HA-truncation/HA-WT-His mRNAs relative to those of the HA-WT/HA-WT-His. Statistical significance was determined by one-way analysis of variance (ANOVA) with Dunnett’s correction for multiple comparisons; asterisks indicate significant differences compared to the wild-type template as follows: **, *P* ＜0.01. (**C**) Dose-dependent effect of HA-WT-His in the presence of 250 ng wild-type or 5´L8 template of WSN in the two-template competition RNP reconstitution system. Increasing concentrations (0, 250, 500, 750, and 1,000 ng) of the pHW-mut-HA-WT-His plasmid were co-transfected with 250 ng pHW-mut-HA (wild-type or 5´L8), together with plasmids expressing WSN viral polymerase subunits (PB2, PB1, and PA-C95A) and NP into HEK-293T cells. Accumulation of HA mRNAs was detected by primer extension analysis at 24 h post-transfection. The graphs display the mean signal intensity of HA-WT-His mRNA (blue) and HA-WT or 5´L8 mRNA (red), each normalized to that of 0 ng pHW-mut-HA-WT-His or pHW-mut-HA. The data in panels **A** to **C** represent the means ± SEM from three independent experiments.

To investigate how the 5′-end ssNCRs affect influenza viral RNA transcription in a multi-template environment, we specifically investigated template competition effects. For this purpose, we established a two-template competition RNP reconstitution system. In this system, the truncation mutant template was co-transfected with a modified wild-type template containing 5 × His tag insertion immediately downstream of the HA signal peptide (HA-WT-His). Both templates were derived from the mutant vRNA-expressing plasmid (pHW-mut-HA). The inserted 5 × His tag sequence in the HA-WT-His mRNA lies upstream of the reverse transcription primer used in primer extension analysis, allowing clear differentiation between the wild-type transcript (with the insertion) and the 5′-end ssNCR truncated transcript (without the insertion). As a control, we first confirmed that the HA-WT-His template produces mRNA at levels comparable to the unmodified HA-WT template in both the previously used single- and multi-template competition RNP reconstitution systems (Fig. 4A).

To specifically examine the viral RNA transcription activity, we also employed a replication-deficient polymerase mutant (PA-C95A) that primarily permits viral RNA transcription only in the RNP reconstitution system (31). As shown in Fig. 4B, upon co-transfection of an equal amount of HA-WT-His and HA-WT or HA truncation mutant vRNA expressing plasmids, the 5′L8 mutant produced significantly reduced mRNA levels compared to that of the wild-type template. To further confirm the regulatory effects of the 5′-end ssNCR on the competitive viral RNA transcription, we carried out a dose-dependent experiment between the wild-type template and 5′L8 template of WSN. The ratios of co-transfected HA-WT-His and HA-WT/5′L8 templates were 0, 1:1, 2:1, 3:1, and 4:1. As shown in Fig. 4C, in the competition with the HA-WT-His template, the competitive ability of the HA-5′L8 template is significantly lower than that of HA-WT template in all cases. Taken together, these results clearly demonstrate that the 5′-end ssNCRs indeed play a role in determining the optimal viral mRNA level of the segment in a template-competitive manner.

### A point mutation G1724A at the seventh nucleotide upstream of the truncation site restores virus growth and compensates for both the transcription and incorporation of the HA segment

**Fig 5:**
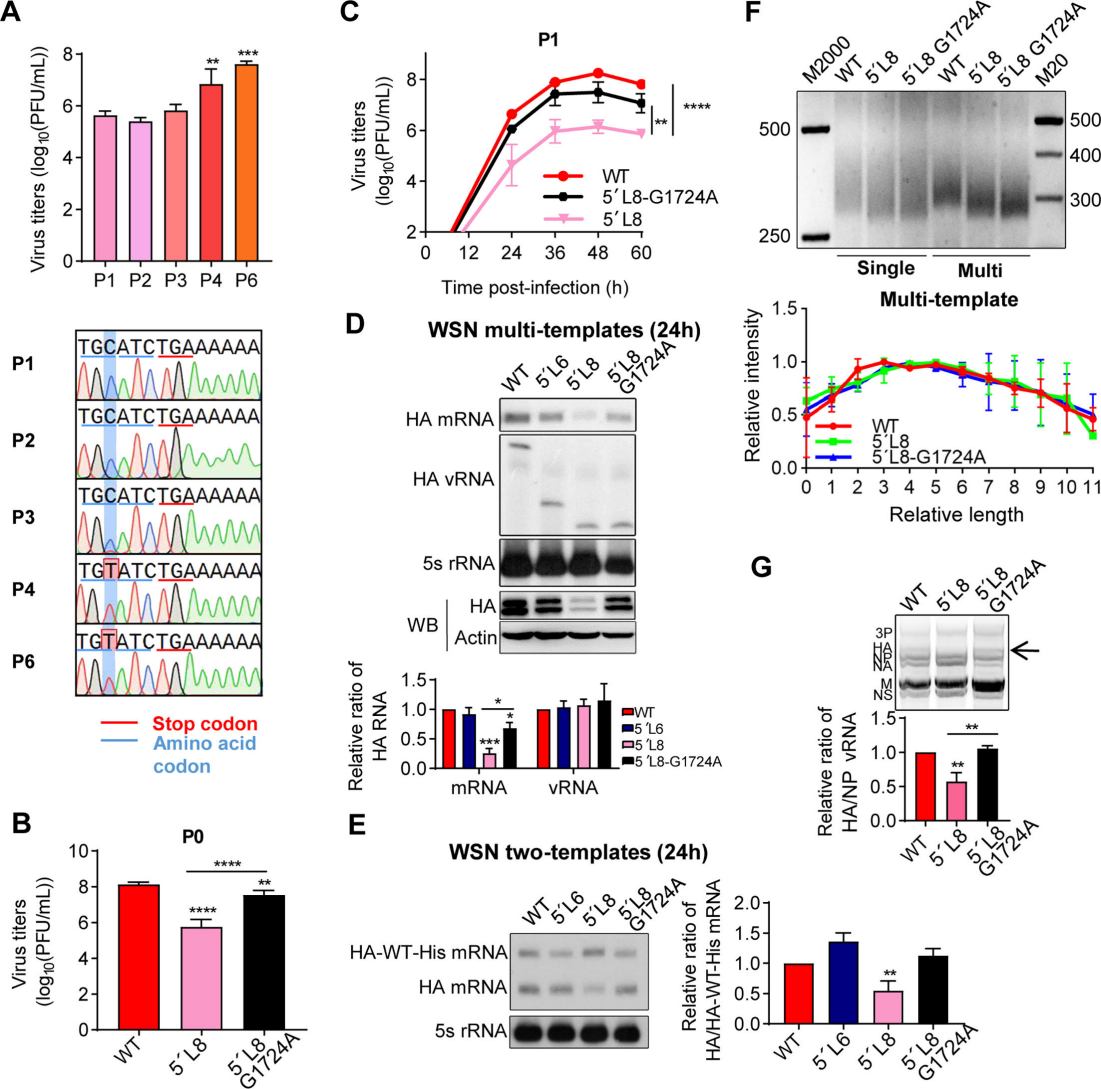
Point mutation G1724A at the seventh nucleotide upstream of the truncation site restores the virus growth and compensates for both the transcription and incorporation of the HA segment. (**A**) Viral titers of the 5´L8 virus passaged in MDCK cells from P1 to P6 (top) and reverse cDNA sequence traces of the 5′ terminus of HA vRNA (bottom). The sequence traces are representative of three independent biological replicates (*n* = 3). (**B**) Viral titers of rescued wild-type, 5′L8, or 5′L8-G1724A viruses (P0). Viruses were rescued in HEK-293T and MDCK co-cultured cells. Supernatants were harvested at 48 h post-transfection, and titers were determined by plaque assay on MDCK cells (P0). (**C**) Growth curves of WSN wild-type, 5′L8, and 5′L8-G1724A viruses infected at an MOI of 0.001 (P1). Viral growth curves were determined by plaque assay on MDCK cells at 24 h, 36 h, 48 h, and 60 h post-infection. Statistical significance for panels **B** and **C** was determined using a two-way analysis of variance with Dunnett’s correction; asterisks indicate significant differences from the wild-type virus as follows: **, *P* ＜0.01; ***, *P* ＜0.001; ****, *P* ＜0.0001. (**D**) Truncation of the 5′-end H1-ssNCR with point mutation G1724A in the WSN multi-template RNP reconstitution system. The plasmids co-transfected into HEK-293T cells are described in Fig. 3B. (**E**) Truncation of the 5′-end H1-ssNCR with point mutation G1724A in the WSN two-template RNP reconstitution system. The plasmids co-transfected into HEK-293T cells are described in Fig. 4B. In both panels **D** and **E**, accumulation of HA RNAs at 24 h-post-transfection was detected by primer extension analysis, and HA protein levels were assessed by Western blot analysis in panel **D**. The graphs display the mean signal intensity of mutant HA RNAs relative to those of the wild-type (**D**) and HA-truncation/HA-WT-His mRNAs relative to those of the HA-WT/HA-WT-His (**E**). (**F**) The length of poly(A) tails for the 5′-end H1-ssNCR truncated templates was assessed in both single- and multi-template RNP reconstitution systems. M2000 and M20 indicate DNA markers. Plasmids co-transfected into HEK-293T cells were as described previously. The 3′-ends of HA mRNAs, derived from HA-WT, 5′L8, and 5′L8-G1724A templates, were detected by PAT assay. As expected, the 3′-end of 5´L8 mRNA is 24 nt shorter, thus migrating faster than that of WT mRNA. The graph displays the relative intensity of each ladder corresponding to wild-type and mutant templates. (**G**) M-RT-PCR analysis of WSN wild-type and mutant vRNA levels in virions (P1). The graph displays the mean signal intensity of mutant HA/NP vRNAs relative to those of the wild-type. Statistical significance for panels **A**, **D**, **E**, and **G** was determined using one-way analysis of variance (ANOVA) with Dunnett’s corrections. Asterisks indicate significant differences from the wild-type template as follows: *, *P*＜0.05; **, *P* ＜0.01; ***, *P* ＜0.001. Data in panels **A** to **G** represent the means ± SEM from three independent experiments.

In order to further investigate the mechanism of the 5′-end ssNCRs on regulating the competitive RNA transcription efficiency during infection, we serially passaged the rescued 5′L8 mutant virus (from P0 to P6) in MDCK cells. We found that the growth rates were increased especially from P4 to P6 and reached maximal titers between 1 × 10^7^ and 1 × 10^8^ PFU/mL, similar to that of the wild-type virus (Fig. 5A, top). We then sequenced the HA vRNA of these viruses (P1–P6). We found that a point mutation G1724A (C1724T in positive sense), a synonymous mutation in the HA open reading frame which is at the seventh nucleotide upstream of the truncation, repetitively appeared in three independent passages. The mutation appeared in P3 at earliest and then gradually became dominant in the subsequent passages (Fig. 5A, bottom).

To confirm the effect of the G1724A mutation on WSN virus growth, we introduced the G1724A mutation into the 5′L8 mutant virus with the reverse genetics and compared the titer of 5′L8-G1724A with those of WT and 5′L8 viruses at P0 and P1. In contrast to the significant attenuation (> 2 log reduction) observed for the original 5′L8 mutant, the 5′L8-G1724A mutant showed a dramatically increased titer to the similar level of WT at both P0 and P1 (Fig. 5B and C). These results show that the adaptive mutation G1724A in the open reading frame is indeed responsible for reversing the attenuation 5′L8 virus during the serial virus passages.

We then examined the 5′L8-G1724A mutant using both the multiple-template and two-template competitive RNP reconstitution systems described above. As expected, introduction of the adaptive G1724A mutation into the 5′L8 mutant significantly enhanced its competitiveness in both systems (Fig. 5D and E). Given that the 5′L8 truncation is located very close to the U stretch, which is well known to serve as the template for viral mRNA polyadenylation, we employed an RT-PCR poly(A) test (PAT) assay (32) to assess whether the mutation affects HA mRNA poly(A) tail length. Since poly(A) tail length can influence mRNA stability and translation efficiency (33, 34), we analyzed HA mRNAs generated in both single-template and multi-template RNP reconstitution systems. PAT assay results showed that the poly(A) tail lengths of wild-type, 5′L8, and 5′L8-G1724A mRNA were similar (Fig. 5F), indicating that the restored HA mRNA levels were not due to changes in polyadenylation. In addition, we assessed genomic RNA content in virions rescued from 5′L8-G1724A virus using M-RT-PCR analysis (Fig. 5G). The results revealed that HA segment packaging efficiency was significantly improved in the 5′L8-G1724A virus, reaching levels comparable to those of the wild-type. In conclusion, the adaptive mutation restores viral growth by compensating for both HA mRNA reduction in a multi-segment context and the HA vRNA packaging defect in virions.

### The secondary structure around the 5′-end H1-ssNCR may contribute to a dual role in competitive transcription and selective packaging

We recently investigated the structural features of the IAV at single-nucleotide resolution by using icSHAPE and PARIS experiments (Sequence Read Archive under Bioproject number PRJNA1080059 and accession numbers SRR28084371, SRR28084636, SRR28084670, and SRR28084637) (35) both *in vivo* and *in virio*. By combining these data sets with secondary structure predictions of vRNA termini using the RNA-fold WebSevers (http://rna.tbi.univie.ac.at/cgi-bin/RNAWebSuite/RNAfold.cgi), we identified a local RNA structure formed by the 5′-terminal coding region and the 5′-end ssNCR, surrounding the U stretch. In this model, the U stretch resides within a large single-stranded loop.

**Fig 6:**
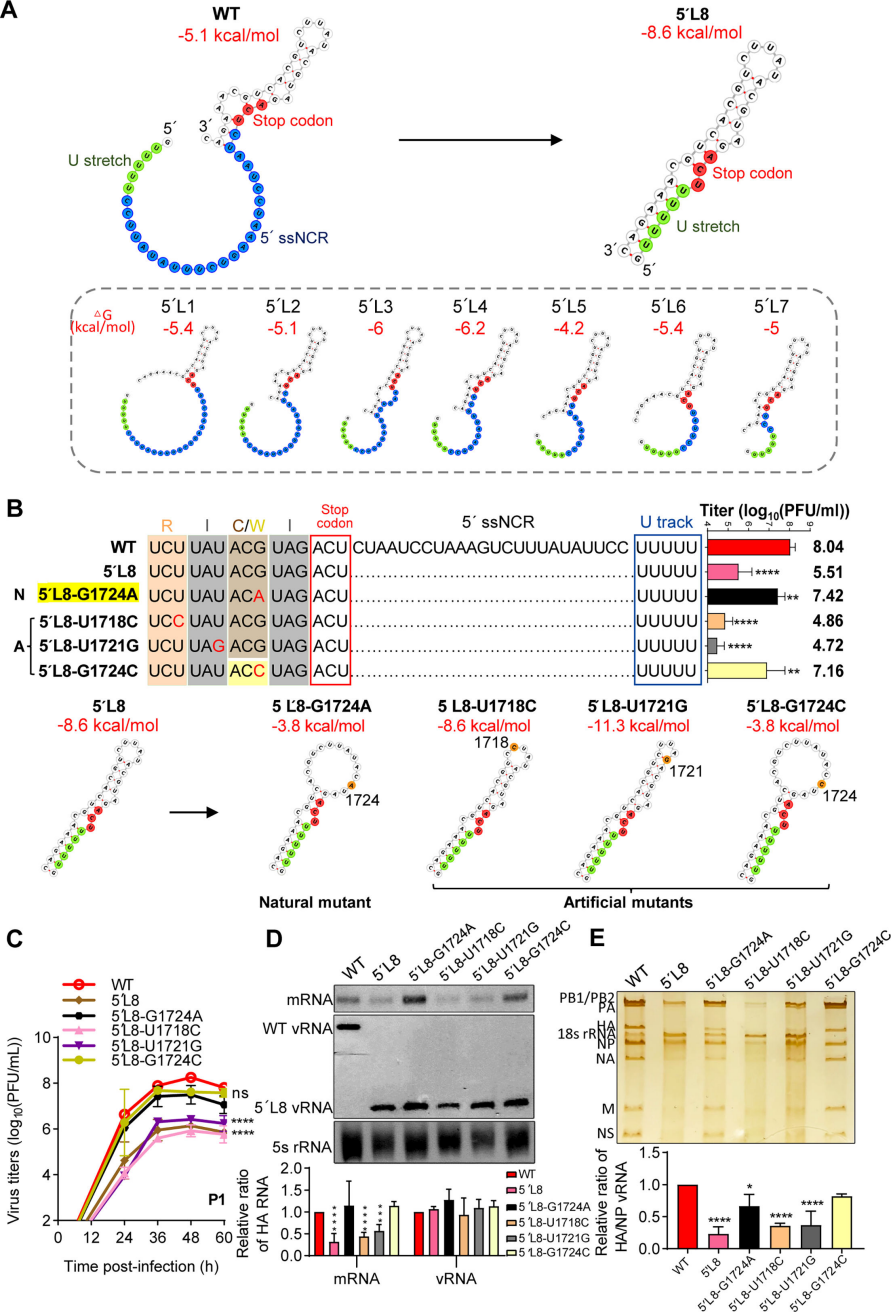
The secondary structure around the 5′-end H1-ssNCR may contribute to a dual role in competitive transcription and selective packaging**.** (**A, B**) Predicted secondary structures of the 5′-terminal open reading frame (ORF), segment-specific non-coding region (ssNCR), and U stretch of wild-type HA and 5′ L1–5′L8 mutants (**A**), as well as 5´L8 with introduced point mutations (**B**). Red nucleotides indicate stop codons. The ssNCRs and U stretches are shown in blue and green, respectively. The point mutations in 5′ L8 variants are highlighted in orange. (**B**) Sequences and titers of the rescued WSN wild-type and mutant viruses. N: natural mutant. A: artificial mutants. The flesh pink, gray, and brown/yellow boxes indicate individual amino acids upstream the stop codon. The stop codons and U stretches are highlighted in red and blue frames, respectively. The graph on the right side displays the mean titers of wild-type and mutant viruses in P0. (**C**) Growth curves of mutant viruses infected at an MOI of 0.001 (P1). Viral growth curves were determined by plaque assay on MDCK cells at 24 h, 36 h, 48 h, and 60 h post-infection. Statistical significance for panels **B** and **C** was determined using a two-way analysis of variance with Dunnett’s correction for multiple comparisons; asterisks indicate significant differences from the wild-type virus as follows: **, *P* ＜0.01; ****, *P* ＜0.0001. (**D**) 5´ L8 variants with introduced point mutations in the WSN multi-template RNP reconstitution system. The plasmids co-transfected into HEK-293T cells are described in Fig. 3B. Accumulation of HA RNAs was detected by primer extension analysis. The graph displays the mean signal intensity of mutant HA RNAs relative to those of wild-type RNAs. (**E**) Analysis of the packaging efficiency of each individual vRNA with silver-staining gel. Wild-type and mutant viruses were amplified on MDCK cells and purified by ultracentrifugation through a 30% sucrose cushion. Then, the virion vRNAs were extracted and analyzed on a 2.8% denaturing polyacrylamide gel, followed by silver staining visualization. The graph displays the mean signal intensity of mutant HA/NP vRNAs relative to those of the wild-type RNAs. Statistical significance for panels **D** and **E** was determined using one-way analysis of variance with Dunnett’s correction for multiple comparisons; asterisks indicate significant differences from the wild-type template as follows: *, *P*＜0.05; ***, *P* ＜0.001; ****, *P* ＜0.0001. The data in panels **B** to **E** represent the means ± SEM from three independent experiments.

We further predicted the secondary structures of the 5′L1 to 5′L8 truncation mutants using the same tool. Interestingly, in the 5′L1 to 5′L7 mutants, the U stretch remained within a progressively smaller single-stranded loop, whereas in the 5′L8 mutant, it shifted into a base-paired duplex region (Fig. 6A). Correspondingly, the minimum free energy (MFE) values of the 5′L1 to 5′L7 structures hovered around −5 kcal/mol, similar to the wild-type. In contrast, the MFE of 5′L8 decreased to −8.6 kcal/mol (Fig. 6A), indicating increased structural stability. This marked structural alteration in 5′L8 aligns with its functional defects in competitive transcription and selective packaging, as observed in our experiment.

To further investigate the mechanism by which the G1724A mutation rescues these defects, we analyzed its impact on the RNA structure. Structural predictions revealed that the G1724A mutation enlarged the loop near the U stretch, shifting nucleotides out of the duplex and restoring a more flexible configuration. The MFE of this new structure increased to –3.8 kcal/mol– higher (less stable) than even the wild-type, suggesting that the mutation disrupts over-stabilization and restores transcriptional and packaging compatibility (Fig. 6B). This significant structural change of 5′L8 is consistent with its phenotype in competitive transcription and selective packaging, as we observed.

To further dissect the contribution of the local sequence context to the structural and functional rescue conferred by the G1724A mutation, we introduced three additional point mutations: two silent mutations (U1718C and U1721G), located at the third nucleotide position of two codons upstream of the truncation site and one nonsynonymous mutation (G1724C) at the same position as natural adaptive mutation (G1724A). RNA secondary structural predictions revealed that U1718C and U1721G mutations resulted in structures similar to that of the 5′L8 mutant, with significantly lower MFE. In contrast, the G1724C mutation generated a structure and MFE nearly identical to that of G1724A (Fig. 6B). We next attempted to rescue viruses carrying these mutations and measured viral titers at P0 and P1. In parallel, genomic RNAs from P1 virions were analyzed via silver staining to assess the packaging efficiency. Additionally, we performed multi-template RNP reconstitution assays to evaluate their transcriptional competitiveness. The results showed that the two silent mutations, U1718C and U1721G, caused a significant reduction in both P0 and P1 virus titers (Fig. 6B and C) and impaired transcriptional competition (Fig. 6D). In contrast, the G1724C mutant displayed viral titers and transcriptional activity comparable to those of the G1724A mutant (Fig. 6B–D). Notably, HA segment incorporation levels in the mutant virions closely resemble their viral titers and mRNA expression in the multi-template context (Fig. 6E). Collectively, these findings support the conclusion that the secondary structure surrounding the 5′-end H1-ssNCR may play a critical role in both competitive transcription and selective packaging of the HA segment and that this function is highly sensitive to the local sequence and structural context.

## DISCUSSION

The 3′ and 5′ segment-specific NCRs of influenza A virus are highly conserved in both sequence and length across the same segment in different influenza A viruses. Despite this conservation, the precise biological roles of the ssNCRs in regulating influenza A virus replication have remained incompletely understood. In addition to serving as parts of the packaging signal, we previously reported that the 3′-terminal noncoding regions (3′-end ssNCRs), together with their adjacent coding sequences, act synergistically to ensure optimal levels of HA vRNA replication in a multi-segment context during infection (28). In this study, we extended this investigation to the 5′-end ssNCRs through a series of truncation experiments. Interestingly, in the context of H1N1 virus infection, we observed that truncations in the 5′-end ssNCR, specifically between the U stretch and the stop codon, impacted both HA vRNA packaging and mRNA transcription, while having minimal effect on HA vRNA replication under competitive, multi-template conditions. This finding contrasts with the role of the 3′-end ssNCRs, which primarily influence HA vRNA replication (28). Furthermore, we identified an adaptive mutation occurring consistently during virus passaging, located seven nucleotides upstream of the truncation site within the coding region. This mutation was sufficient to significantly restore HA mRNA expression and vRNA packaging, ultimately rescuing viral replication. Our structural analyses suggest that the RNA secondary structure surrounding the 5′-end H1-ssNCR contributes to this dual function, potentially by modulating both transcription efficiency and selective genome packaging. Based on these findings, we propose that the 5′-end ssNCRs play a dual regulatory role during infection by fine-tuning the segment-specific mRNA expression and simultaneously serving as essential elements of the packaging signal. This highlights the intricate coordination between replication, transcription, and packaging in influenza A virus biology.

In the double-helical conformation of vRNP, the terminal 3′ and 5′ ends of the viral RNA are joined by a short duplex region, formed by conserved promoter sequences, which interacts directly with the viral RNA polymerase. The remainder of the vRNA is wrapped around the helical backbone composed of nucleoprotein. The currently favored model suggests that secondary or tertiary RNA structures of vRNAs can protrude from the surface of the vRNP, contributing to the functional specificity (17, 22, 36). In support of this model, the 3′ and 5′-end ssNCRs have been implicated as bundling signals that mediate selective genome packaging (25). Multiple studies have shown that truncations in the 3′ and/or 5′-end ssNCRs impair the packaging efficiency of corresponding IAV segments (27, 37). Consistent with these findings, we observed that truncation of the 5′-end ssNCRs resulted in impaired packaging of the HA segment. Importantly, our study also reveals a previously unrecognized regulatory function. Beyond packaging, the 5′-end ssNCR truncation mutant exhibited significantly reduced the HA mRNA level specifically in a multi-template competitive environment. These results identify a dual role for the 5′-end ssNCRs in regulating selective packaging and competitive transcription of influenza A virus.

In terms of the 3′ and 5′-end ssNCRs in regulating viral RNA syntheses, we previously revealed that the 3′-end ssNCRs mainly play an important role in determining the template preference on viral RNA replication, while, in this study, we further demonstrated that the 5′-end ssNCRs specifically mediate the template preference on viral RNA transcription. Interestingly, we found that a long-range interaction between the 3′-end H1-ssNCR and its adjacent ORF (20–40 nt downstream) is particularly involved in the regulation of competitive vRNA synthesis; by contrast, a local RNA structure formed by 5′-end H1-ORF and -ssNCR around the U stretch is implicated in the regulation of the optimal viral mRNA synthesis. We speculate, that 3′-end secondary RNA structures may mediate viral RNA polymerase processivity during viral RNA replication, particularly at the initiation stage. In contrast, the local structures formed around the 5′-end H1-ssNCR may play a role in competitive transcription, most likely at the termination stage. These results are in line with the findings that no stringent compatibility is required between the 3′-end and 5′-end ssNCRs and that 3′-end ssNCRs play a more important role than 5′-end ssNCRs in affecting virus growth (27, 28).

Considering the effects observed upon truncations of the 3′- and 5′-end ssNCRs in both single- and multiple template RNP reconstitution systems, it can be inferred that competition between segments is absent in the single-template system but present in a multi-template environment. Distinct RNA secondary structures likely form at the 3′- and the 5′- termini of each segment, conferring segment-specific preferences that influence competitive viral RNA replication and/or transcription. Moreover, during the course of influenza virus infection, it is well established that the expression of viral proteins follows a temporal pattern. Some viral proteins are produced at early stages (e.g., PB2, PB1, PA, NP, and NS1), whereas other viral proteins are expressed at relative late stages. This temporal expression is segment-specific and correlates with the relative abundance of their corresponding mRNA (14, 15). However, the regulatory mechanism underlying this expression pattern remains largely undefined. Based on our findings, we propose that both the 3′ and 5′-end ssNCRs contribute either directly or indirectly to the regulation of segment-specific transcription dynamics. Further studies will be required to test this hypothesis and to elucidate the underlying molecular mechanism.

Since we found that changes in the MFE of secondary structures around the 5′-end H1-ssNCR closely correlated with virus titers, mRNA expression levels, and vRNA incorporation efficiency, we speculated that the U stretch within the duplex region plays a critical role in determining structural alterations, which, in turn, influence both transcription and packaging processes. To further explore this, we analyzed the RNA structures containing the U stretch in the HA segment of other influenza strains (e.g., CA04/H1N1, CAIV/H2N2, and H5N1), as well as in other genomic segments of the WSN strain. In most cases, the U stretch was located within a single-stranded region of the local RNA structure. When the U stretch was involved in base pairing, the corresponding RNA structures typically exhibited relatively low MFEs with increased stability. Considering that the virus is unlikely to acquire an insertion of several consecutive bases during adaptation, we infer that the adaptive mutation G1724A increases the MFE and enlarge the loop adjacent to the U stretch. This alteration reduced local RNA stability to a level even lower than that of the wild-type structure, thereby restoring the regulatory function of the region in competitive transcription and selective packaging.

In summary, the 5′-end ssNCRs of influenza vRNAs not only act as selective packaging signals but also play a key role in modulating competitive viral RNA transcription efficiency. The dual regulatory role is likely governed by a secondary structure surrounding the 5′-end ssNCRs during infection. Our findings further enhance the current understanding of the functional importance of noncoding regions in the influenza virus genome, particularly in coordinating viral RNA synthesis and selective genome packaging. These insights may offer alternative antiviral targets and inform novel strategies for manipulating viral protein expression and progeny virion assembly, with potential applications in the development of more effective influenza vaccines.

## MATERIALS AND METHODS

### Cells, viruses, and plasmids

Human embryonic kidney 293T (HEK-293T) cells and Madin-Darby canine kidney (MDCK) were cultured in Dulbecco’s modified Eagle medium (DMEM) supplemented with 10% fetal bovine serum (FBS). Cells were maintained at 37°C and 5% CO_2_. Plasmids pcDNA-PB2, pcDNA-PB1, pcDNA-PA, pcDNA-NP, and pcDNA-PB1a, for the single-template RNP reconstitution system of influenza A/WSN/33 (H1N1) virus (30, 38), have been described previously. Recombinant influenza A/WSN/33 (H1N1) virus was generated using the pHW2000 eight-plasmid system (39). To construct the pHW2000-mut plasmid to express vRNA but not mRNA, the pHW2000 plasmid was modified by removing the truncated immediate-early promoter of the human cytomegalovirus (CMV). Plasmids pHW2000-mut-HA-5′L1 to pHW2000-mut-HA-5′L8 were generated from pHW2000-mut-HA-WT by PCR amplification of the HA sequence with corresponding primers, introducing truncations in the 5′-end H1-ssNCR. Plasmids pHW2000-mut-NA-5′Ld were generated from pHW2000-mut-NA-WT by PCR amplification of the NA sequence with corresponding primers, introducing complete truncation in the 5′-end N1-ssNCR. The PCR products were then ligated into the BsmBI site of the pHW2000 vector. Plasmid pHW2000-mut-HA-WT-His and plasmids pHW2000-mut-HA-5′L8 with point mutations were generated from the plasmids pHW2000-mut-HA-WT and pHW2000-mut-HA-5′L8, respectively, using site-directed PCR mutagenesis.

### Reverse genetics

The recombinant influenza A/WSN/33 (H1N1) (WSN) virus was generated using the pHW2000 eight-plasmid system (39). Approximately 7 × 10^5^ HEK-293T and 4 × 10^5^ MDCK co-cultured cells were transfected with 0.5 µg each of pHW2000-PB2, pHW2000-PB1, pHW2000-PA, pHW2000-mut-HA (wild-type or truncation mutant), pHW2000-NP, pHW2000-NA, pHW2000-M, and pHW2000-NS using Lipofectamine 2000 and Opti-MEM according to the manufacturer’s instructions. At 24 h post-transfection, DMEM containing 10% FBS was replaced with DMEM containing 0.5% FBS, 0.5 µg/mL TPCK trypsin, and 100 U/mL penicillin-streptomycin (ThermoFisher). The virus supernatants were harvested at 48 h post-transfection. For serial passaging, A/WSN/33 wild-type and mutant viruses were used to infect MDCK cells at a multiplicity of infection (MOI) of 0.001. The virus titers were examined by plaque assay.

### Virus growth curve

MDCK cells were infected with either wild-type WSN or recombinant virus at an MOI of 0.001. At 24, 36, 48, 60, and 72 h post-infection, the supernatants were collected. The virus titers were determined by plaque assay on MDCK cells.

### Multiple RT-PCR analysis of virion and cellular RNAs

Approximately 7 × 10^5^ HEK-293T and 4 × 10^5^ MDCK co-cultured cells were co-transfected with 0.5 µg of each plasmid used in reverse genetics. The virus supernatants were harvested at 48 h post-transfection. In the single-cycle rescued system, the cells were cultured in DMEM with 5 mM NH_4_Cl at 6 h post-transfection to prevent multi-cycle infection and were harvested at 48 h post-transfection. The RNAs were extracted using TRI LS Reagent for virions or TRI Reagent for cells (Sigma-Aldrich). The RNAs were dissolved in 20 µL (virions) or 30 µl (cells) of nuclease-free water. Approximately 500 ng of RNAs was reverse-transcribed by SuperScript III (Invitrogen) with universal influenza A virus reverse transcription (RT) primers (5′-AGCAAAAGCAGG-3′ and 5′-AGCGAAAGCAGG-3′). The RT products were then amplified by multi-segment reverse transcription-PCR (M-RT-PCR) using a pair of primers to target all vRNAs as previously described (Uni12/Inf-1: 5′-GGGGGGAGCAAAAGCAGG-3′ and Uni13/Inf-1: 5′-CGGGTTATTAGTAGAAACAAGG-3′) (29). The PCR products were then analyzed by 1.5% agarose gel in Tris acetate-EDTA buffer and visualized by ethidium bromide staining.

### Hemagglutination assay

Rescued recombinant WSN viruses were harvested at 48 h post-transfection, as described previously. The viruses were serially diluted and then incubated with 1% guinea pig red blood cells at room temperature for 40 min before reading the results. The highest dilution that did not cause cell agglutination was recorded as the hemagglutination titer of the virus.

### Western blot analysis

In single-/multi-template RNP reconstitution systems, cells were harvested for Western blot analysis at 24 h post-transfection. Cells were lysed with Cytobuster lysis buffer (Novagen) and denatured at 95°C. In the rescued system, supernatants were harvested for Western blot analysis at 48 h post-transfection and directly denatured at 95°C. Both samples were then subjected to Western blotting with a monoclonal anti-HA antibody, with anti-actin, anti-NP (cells), and anti-M1 (supernatants) antibody as an internal control.

### RNP replicon systems and primer extension analysis

For single-template RNP reconstitution system, vRNPs were reconstituted by transiently transfecting approximately 10^6^ HEK-293T cells with 0.5 µg each of pcDNA-PB2, pcDNA-PB1/pcDNA-PB1a, pcDNA-PA, pcDNA-NP, and pHW2000-mut-HA (wild-type or truncation mutants) using Lipofectamine 2000 and Opti-MEM according to the manufacturer’s instructions. For the multi-template RNP reconstitution system, pHW2000-PB2, -PB1, -PA, -mut-HA (wild-type or truncation mutants), -NP, -NA and -NS plasmids or pHW2000-PB2, -PB1, -PA, -HA, -NP, -mut-NA (wild-type or truncation mutant), and -NS plasmids were transfected into approximately 10^6^ HEK-293T cells. Plasmids pcDNA-PB1a and pPOLI-PB1 encoding polymerase with active site mutations (D445A/446A) were used as the negative control (30). For the two-template and dose-dependent competition RNP reconstitution system with a replication-deficient but transcription-competent polymerase (PA-C95A) (31), approximately 10^6^ HEK-293T cells were transfected with 0.5 µg each of pcDNA-PB2, pcDNA-PB1/pcDNA-PB1a, pcDNA-PA-C95A, pcDNA-NP, and pHW2000-mut-HA (wild-type-His and wild-type/truncation mutants). Cells were harvested at 24 h post-transfection. Total RNA was extracted using TRI reagent (Sigma-Aldrich) and dissolved in 30 µL of nuclease-free water. RNA was analyzed by primer extension using ^32^P-labeled primers specific for negative- or positive-sense viral RNAs as well as 5S rRNA. The primers for segment 4 or 6 negative- or positive-sense RNA of WSN virus and 5S rRNA have been described previously (40). 5S rRNA was used as an internal loading control. Primer extension products were analyzed by 12% denaturing PAGE with 7 M urea in Tris-borate-EDTA (TBE) buffer and visualized by phosphor-imaging on an FLA-5000 scanner (Fuji). ImageJ was used to analyze the ^32^P-derived signal (41).

### RNA sequencing

The RNA of the virus stock was extracted with TRI LS Reagent (Sigma-Aldrich) and reverse-transcribed using the SuperScript III first-strand synthesis system (Invitrogen) with universal influenza A virus reverse transcription (RT) primers for multiple RT-PCR analysis. The RT products were then amplified by PCR using HA segment-specific primers (Fwd_5′-TGAGTAGAGGGTTTGAGTCCGG-3′; Rev_5′-ACGCGTGATCAGTAGAAACAAGG-3′), followed by sequencing of the PCR products.

### PCR poly(A) test (PAT) assay

Approximately 10^6^ HEK-293T cells were co-transfected with a single-template RNP reconstitution system and multi-template RNP reconstitution system, as mentioned above (32). Cells were harvested at 24 h post-transfection. Total RNAs were extracted using TRI Reagent (Sigma-Aldrich) and dissolved in 30 µL of nuclease-free water. A barcoded RNA (5′-P-CCUAUAGUGAGUCGUAUUAA-ddC-3′) was linked to the 3′-end of cellular RNAs with T4 RNA ligase 1 (New England Biolabs). The HA mRNA poly(A) length was measured by reverse transcription of total cellular mRNAs with the barcoded primer targeting poly(A) tail (5′-TTAATACGACTCACTATAGGTTTTT-3′), followed by a PCR with an HA-specific primer (5′-TGGAAAGTGTAAGAAATGGG-3′) and the barcode primer (5′-TTAATACGACTCACTATAGGTTT-3′). The PCR products were then analyzed by 3% agarose gel in Tris acetate-EDTA buffer and visualized by ethidium bromide staining. The product ladders were equally divided into 12 fractions, and ImageJ was used to analyze the intensity of ladders. The brightest fraction of each lane was set as 1.

### Silver staining of virion RNAs

MDCK cells in the T75 culture flask were infected with wild-type WSN virus or mutant viruses at an MOI of 0.001. Viral supernatants were harvested at 48 h post-infection and clarified by centrifugation at 3,000 rpm for 30 min. The supernatant was further centrifuged at 10,000 rpm for 1 h at 4°C. Then, the clarified supernatant was collected and loaded onto a 30% sucrose TNE buffer and further centrifuged at 25,000 rpm for 2.5 h at 4°C. The RNAs of the virus pellets were extracted using the TRI LS reagent (Invitrogen) and separated on a 2.8% denaturing polyacrylamide gel containing 7 M urea. The gel was stained with a SilverQuest silver-staining kit (Invitrogen). The intensities of RNA bands were quantified using ImageJ software.

### Statistics

GraphPad Prism software, version 8, was used for statistical analysis. Two-way analysis of variance (ANOVA) with Dunnett’s correction and Student’s t test were used for two-variable comparisons, while one-way analysis of variance (ANOVA) with Dunnett’s correction was used for one-variable comparisons. *P* values of ＜0.01 were considered to be significant.

## Data Availability

All data supporting the conclusions of this study are available from the corresponding author upon reasonable request.
